# Enhancing EFL students’ engagement in online synchronous classes: The role of the Mentimeter platform

**DOI:** 10.3389/fpsyg.2023.1127520

**Published:** 2023-02-23

**Authors:** Ayat Tarazi, José Luis Ortega-Martín

**Affiliations:** ^1^Department of Didactics of Languages and Literature, University of Granada, Granada, Spain; ^2^Department of Didactics of Languages and Literature, Faculty of Educational Sciences, University of Granada, Granada, Spain

**Keywords:** Mentimeter platform, student engagement, synchronous education, online synchronous classes, teachers’ perspectives

## Abstract

The present research aimed to evaluate teachers’ attitudes toward the use of the Mentimeter platform in synchronous education. This study also sought to delve into teachers’ perspectives on the role of the Mentimeter platform in improving students’ engagement in online synchronous classes. To that end, the study was carried out in Palestine in response to the ongoing shift to synchronous online teaching to raise teachers’ consciousness about the importance of using online tools to boost students’ engagement and make them feel connected in synchronous classes. A quantitative approach was used to collect data, and 44 Palestinian teachers from various educational institutions completed closed-ended questionnaires. The study’s outcomes demonstrated that teachers had positive attitudes toward employing the Mentimeter platform in synchronous education. The results also indicated that almost all educators perceived that the Mentimeter platform serves a vital role in increasing student engagement in online synchronous sessions. Teachers asserted that Mentimeter presentation styles decrease the probability of boredom among students, which in turn encourages them to actively participate in online synchronous classes. Enhancing students’ engagement in online synchronous classes was still a main challenge for educators. Therefore, to increase students’ involvement in synchronous learning environments, it is necessary to regularly train the teaching staff of schools, learning institutions, and colleges in the use of Mentimeter. Future studies in Palestine and other countries are also recommended to simultaneously concentrate on teachers’ and students’ viewpoints.

## Introduction

The success of synchronous education highly depends on the degree of student engagement in classroom contexts. According to Martin and Bolliger (2018), engagement serves a crucial role in fostering online learning. Likewise, Britt (2015) highlighted the significance of student engagement in online synchronous classes by referring to its enormous impact on students’ intellectual growth. Student engagement is essential to addressing the problems of student loneliness, failure, retention, and boredom in online learning environments (Pawlak et al., 2021; Derakhshan et al., 2022). Student engagement has been generally defined as the level of focus, interest, and enthusiasm that students exhibit to initiate and continue the learning process (Bundick et al., 2014). According to Bower et al. (2015), synchronous education involves students and teachers interacting in a particular virtual environment via an online platform. Weitze (2015) characterized synchronous education as a new educational mode that has a significant impact on pedagogical design.

To increase student engagement with the online educational curriculum, online instructors must devote sufficient time to the search for interactive learning resources (Abrami et al., 2011; Banna et al., 2015; Firman and Rahayu, 2020; Derakhshan et al., 2021). For this purpose, they also need to employ an efficient online platform (Revere and Kovach, 2011). It is up to teachers to design a cooperative learning environment that motivates students to put more effort into learning tasks (Carolan and Kyppö, 2015). There are numerous problems and difficulties with synchronous online education, notably when it comes to student engagement (Rusakova et al., 2022). Most synchronous educational settings still lack personal contact and interaction between teachers and students (Ramsey et al., 2016). Synchronous instruction raised the demand for teachers to be technologically competent (Herawati et al., 2022). From the instructors’ perspectives, synchronous settings necessitate fundamental modifications to the instructors and their instructional techniques (Cain, 2015; Ramsey et al., 2016). Additionally, given the fact that the quality of online instruction is somewhat dependent on teachers’ technological literacy (Bower et al., 2015), teachers need to actively learn how to work with the technology (Grant and Cheon, 2007; Weitze et al., 2013).

One way to facilitate active learning in synchronous educational settings is the implementation of “Audience Response Systems” (ARS). These systems are easily applicable in synchronous educational environments since they enable teachers to offer a variety of questions to students during lectures (Compton and Allen, 2018). The use of such electronic response systems has been shown to be useful in improving student engagement (Morrison, 2015). These days, different digital platforms such as Mentimeter, Kahoot, Plickers, GoSoapBox, and Poll Everywhere make it possible for teachers to boost their students’ engagement in synchronous learning environments (Pikhart et al., 2022a,b). In the current study, researchers aim to delve into the role of the Mentimeter platform as one of these digital platforms. According to Little (2019), Mentimeter is a form of “Student Response System” (SRS) that invites students to participate in conversations and debates using their mobile, laptop, or tablet devices. Similarly, Puspa and Imamyartha (2019) maintained that Mentimeter is accessible software that improves dialogue among students. Quang (2018) also argued that Mentimeter promotes collaborative learning by enabling students to share their opinions with teachers and other students. It also provides users with interactive learning opportunities with its attractive presentation of the results (John, 2018).

Given the value of the Mentimeter platform in online educational environments, the current research intends to assess teachers’ attitudes toward the use of the Mentimeter platform in synchronous online education. This research also attempts to evaluate teachers’ viewpoints regarding the role of the Mentimeter platform in enhancing students’ engagement in online synchronous courses. Consequently, the results of this paper will assist Palestinian educators in overcoming difficulties in enhancing students’ engagement in EFL synchronous learning contexts and increasing their perceptions toward using such an online tool in their synchronous classes. Considering the main goals of the study, three research questions were posed as follows:

▀ What is the role of the Mentimeter platform in enhancing Palestinian students’ engagement in online synchronous classes?
▀ To what extent can teachers’ academic degrees, working environments, and the most commonly used Mentimeter’s tool influence their attitudes toward the use of the Mentimeter platform in online synchronous classes?
▀ Is there any significant relationship between teachers’ attitudes toward the use of this platform in online synchronous classes and their perspectives toward the role of the Mentimeter platform in enhancing students’ engagement?

## Literature review

Several previous studies on using the Mentimeter platform for online instruction have been conducted. Technology-integrated learning and online cutting-edge tools play an important role in enhancing students’ different learning styles and stimulating their interest and motivation toward online learning. In this regard, Madiseh et al. (2022) carried out a scoping review to look into how well mental models are incorporated into teaching and learning. According to the study’s conclusions, Mentimeter’s integration into educational settings improved student motivation by encouraging active participation from students, enhancing student-centered pedagogy, and giving prompt feedback for anonymous student responses.

Attempting to maintain students’ engagement, active participation, and critical thinking skills remains one of the most challenging problems that educators face in online synchronous classes. Several researchers have found solutions to this problem by introducing the Mentimeter into online synchronous classes. For example, Anggriani et al. (2022) used a quasi-experimental method to investigate how the problem-based learning model with the integration of a Mentimeter affects improving elementary students’ critical thinking and collaboration skills. In the same context, a recent review about the use of Mentimeter’s quizzes and online surveys as interactive applications to increase students’ engagement during online lectures conducted by Utomo and Utama (2022) recommended the utilization of interactive applications be carefully prepared to ensure good student engagement but not overly used to prevent any possible distractions. In addition, they came to the conclusion that survey and interactive presentation applications are available to improve classroom and online lecture engagement.

Online instructional teaching tools have a significant impact on students’ awareness and behavior concerning autonomous learning. Consequently, a group of university lecturers conducted an evaluation study to assess the educational applications of Mentimeter to encourage students’ engagement and active learning. The inclusive potential of the Mentimeter application was highlighted by qualitative and quantitative data from both students and educators. This is because the application allows participation from a diverse audience with different backgrounds and capacities, ensuring inclusive and equitable education for all and enhancing interaction, collaboration, attention, and engagement (Pichardo et al., 2021). Moreover, a descriptive study of students’ cognitive abilities and responses to problem-based learning using web-based apps such as the Mentimeter application revealed that students had a positive attitude toward learning statistics, were more enthusiastic about their studies, and their understanding and skills in basic statistical concepts were enhanced as a result of using the Mentimeter application (Ahmad and Subekti, 2021).

In fact, synchronous learning is difficult for students of various learning levels. However, the use of online ICT tools facilitates synchronous learning and makes it more appealing to students and their needs. In this sense, students at Oman College responded positively to the incorporation of Mentimeter in the computer science lecture across nine areas, based on a study: First, the application’s simplicity of use. Second, how much the class participates in class. Third, the freedom to express oneself without worrying about looking foolish. The fourth is drive. Fifth, remembering earlier subjects. The sixth is preparing for the upcoming sessions. The seventh is remembering the key points of the conversation. Eighth grade, participation in class, and overcoming boredom by providing instantaneous feedback on learning is the ninth (Quiroz Canlas et al., 2020). Likewise, Iqbal et al. (2021) investigated students’ perspectives by administering a survey to students in higher education to assess the potential of the Audience Response System (ARS) to improve learning, participation and involvement, sociability, and motivation. According to the findings, the use of ARS has a high potential to improve students’ learning, socialization, and motivation, resulting in a more positive attitude, a sense of well-being, and a sense of accomplishment. The potential of Mentimeter was also emphasized by Moorhouse and Kohnke (2020), who found that SRS like Mentimeter offers students a versatile and varied approach to answering using their mobile devices. They thought Mentimeter had the potential for application in EAP/ESP and English-language classes due to its adaptability and limitless student capacity, as it increases students’ active responses, promotes test results, and increases motivation to learn.

Blended learning has been introduced into the educational system and has significantly changed how students acquire, share, engage, access, and consolidate their knowledge. In this respect, a descriptive qualitative study was conducted at the State University of Medan with the goal of describing the requirements of learning media based on blended learning utilizing a Mentimeter application to enhance students’ creative mathematical thinking abilities. The findings revealed that blended learning-based learning materials are required to improve students’ creative thinking capacity through the use of Mentimeter applications (Andriani et al., 2019). Similar to this, Vallely and Gibson (2018) discussed the use of Mentimeter in both lectures and seminars and suggested instructing more students to employ this technology in their group presentations. Encouraging other higher education peers to use Mentimeter or further incorporate it into lectures and seminars in order to boost student engagement and enhance the overall teaching and learning process.

Besides that, Mentimeter’s feature allows teachers to find out about the students’ opinions. Lapshova et al. (2021) investigated the use of the Mentimeter to conduct surveys remotely. It was discovered that using Mentimeter allowed for efficient surveying, automatic results, and feedback on a specific topic, allowing for beneficial activities in the learning environment and fostering students’ professional competence. The significant impact of Mentimeter assessment features in evaluating students’ progress in online synchronous classes was also reported by Mohin et al. (2022) who conducted a case study on students’ perceptions of using Mentimeter and developed a case study on the Mentimeter formative assessment methodology. According to the results, the use of Mentimeter has a favorable effect on students’ attitudes and performance, the learning environment, and technical factors. Additionally, they came to the conclusion that by encouraging active learning, student participation, and enjoyment, the Mentimeter could play a significant role in altering the dynamics of the huge lecture. Mentimeter makes formative assessment more engaging and enjoyable. On the other side, teachers can benefit from using Mentimeter to evaluate students’ comprehension and enhance their own teaching methods.

In the digital age, online tools are critical for language learning. For example, Wong and Yunus (2020) carried out action research to investigate the efficiency of utilizing the Mentimeter platform in developing students’ writing vocabulary. The findings revealed a considerable discrepancy between the pre- and post-test outcomes. Also, a descriptive qualitative study about the impact of Mentimeter on enhancing students’ engagement in the EFL classroom conducted by Sari (2021) revealed that students had a positive perception toward the implementation of Mentimeter in the EFL classroom. However, the majority of students perceive that no reason makes them dislike Mentimeter’s use in the EFL classroom. The findings also demonstrated a strong influence of Mentimeter on the students’ participation in discussion activities and opinion-sharing while learning English. Additionally, Demirci et al. (2021), who employed the Audience Response System in English teaching, carried out another study on the opinions of 10th-grade students regarding the use of Mentimeter in English class. The outcomes showed that using Mentimeter to teach the course had a good impact on how engaged the students were. It has been determined that it improves student engagement, makes them like the class, motivates them, and aids in their ability to concentrate. Furthermore, Mentimeter fosters competition, enabling students to have fun while learning from their mistakes through constructive criticism.

Students’ agreement on using the Mentimeter in their online English classes was examined by a cross-sectional study done by Puspa and Imamyartha (2019), who found that social science students were in agreement with the use of the online application in their classroom, the use of the Mentimeter in their English environment, and the effects of the Mentimeter application on their speaking and writing abilities. They came to the conclusion that Mentimeter is one of the best technologies utilized by English students and that this study offers major teaching methods in its use as a variation in education for social science students.

By concentrating on the students’ growth in various English skills, a mixed-methods approach to determining the students’ perceptions of the process of learning English using the Mentimeter platform was determined. The findings of the observational analysis describe how students participated in expressing their opinions, seeking the lecturer’s and friends’ advice, practicing idiomatic vocabulary, having a chat face-to-face and taking turns, working in discussion groups, and taking quizzes on the lecturer-prepared Mentimeter platform feature page. Students who responded to the questionnaire and interview questions all agreed that using the Mentimeter platform application helped them learn the English language better. The Mentimeter application’s user viewpoints on English learning have a significant impact on improving English, especially in speaking and vocabulary, and can encourage them to take part in learning so that learning integrated with technology becomes very much needed to be carried out on a sustainable basis (Samad and Munir, 2022).

## Methods

### Participants

Using random sampling, to do so, researchers posted an online survey, an invitation letter outlining the study’s goals and who was eligible to participate, and a consent form on the websites of Palestinian colleges, universities, schools, and educational institutions, as well as on social media sites. A total of 44 Palestinian teachers with different academic degrees were selected from various educational institutions. The final sample comprised 27 males and 17 females. The demographic information of the participants is exhibited in Supplementary Figures 1-3.

The majority of participants (68.2%) were recruited from schools (*N* = 15) and universities (*N* = 15). The rest (31.8%) were selected from colleges (*N* = 7) and educational centers (*N* = 7).

While, the majority of respondents (80%) held MA (*N* = 20) or Ph.D (*N* = 15) degrees. Other respondents (20%) (*N* = 9) had a BA degree.

On the other hand, Supplementary Figure 3 illustrates that the highest frequency of the study sample was 22, with a 50% percentage in favor of all the above, followed by the word clouds tool with a frequency of 8 and an 18.2% percentage, presentations with a frequency of 7 and a 15.9% percentage, open-ended questions with a frequency of 4 and a 9.1% percentage, and the lowest tool, quizzes, with a frequency of 3 and a 6.8% percentage.

### Instrument

To collect the needed data, a researcher-made survey was distributed among respondents. The survey was designed and developed based on the research questions and previous related studies. The developed survey was sent to two experts at Al Quds Open University to validate the accuracy of the items.

### The variables of the study

The variables in this paper were both independent and dependent. The academic degree, type of educational institution, and the most commonly used Mentimeter’s tool were all independent variables. The dependent variables were teachers’ attitudes and perspectives on the role of the Mentimeter platform in enhancing online engagement in synchronous online teaching and teachers’ perspectives on the role of the Mentimeter platform in enhancing online engagement in online synchronous classes.

The researchers designed and developed the questionnaire in English based on the study questions and related studies. The survey comprised three parts; the first part is background information about teachers. The second part examines teachers’ attitudes toward the role of the Mentimeter platform in enhancing online engagement in synchronous online teaching through various statements, such as the ability of this online tool to foster active discussion, be appropriate for different learning styles, be attractive and simple to use in synchronous classes, and the ability and level of students to engage with learning materials and their teachers in different ways. The third part is about teachers’ perspectives on the role of the Mentimeter Platform in enhancing online engagement in synchronous online classes. For example, statements like “students easily engage in open-ended discussion,” “Mentimeter enables more active participation from students in online lectures,” “spontaneous participation is facilitated by Mentimeter’s quiz feature,” and “students’ achievement of the unit learning outcomes was enhanced by the use of Mentimeter presentations” are also measured in this study.

### Procedure

A consent form that guarantees participants’ understanding of the ethical issues regarding voluntary participation, data security, and the anonymity of any data or information used in any publication arising from this study was first distributed to 87 Palestinian teachers. The final number of teachers who indicated their agreement by signing the consent form to participate and have experience using Mentimeter in synchronous classes was (*N* = 44). Having collected the required data, the researchers analyzed the teachers’ viewpoints using the Statistical Package for the Social Sciences (SPSS). The Pearson correlation test was employed to evaluate the relationship between teachers’ attitudes toward the role of the Mentimeter platform in student engagement and their perspectives toward the use of this platform in online synchronous classes. Moreover, using independent t-tests, one-way ANOVAs, and Sheffee tests, the impact of situational variables, including academic degree and working environment, on teachers’ use of the Mentimeter platform in online synchronous classes was examined.

To answer the first research question, the researchers measured mean differences and SD differences between repeated measures with the same instrument for each dimension and the total degree to determine the role of the Mentimeter platform in enhancing students’ online engagement in synchronous education. The researchers carried out a one-way ANOVA test to calculate differences in the total degree of the tool based on the academic degrees of participants for the second research question. To test the role of the working environment (an educational institution), the researchers used Mean and one-way ANOVA to measure the differences in the total degree of the tool. In addition, the researchers used mean, one-way ANOVA, and Scheffe’s *post hoc* test to indicate the differences in the total degree of the tool in order to test the most commonly used Mentimeter’s tool variable. Also, to answer the third research question, the researchers used the Pearson Correlation Test to find out the correlation between teachers’ attitudes toward using the Mentimeter platform in synchronous online teaching and their perspectives toward the platform’s role in enhancing students’ online engagement in synchronous teaching settings.

### Data analysis

The researchers reviewed the output of the questionnaire before entering it into the computer for data analysis. As all instructors’ responses were between “strongly disagree” and “strongly agree,” the researchers then converted these results into numbers (i.e., a score). Table 1 shows the equivalence.

**TABLE 1:** Equivalence of labels.

Label	Score
Strongly disagree	1
Moderately disagree	2
Slightly disagree	3
Slightly agree	4
Moderately agree	5
Strongly agree	6

Also, responses based on estimation averages were scored on a 6-point Likert scale. The correction codes are shown in Table 2.

**TABLE 2:** Correction codes.

Impact degree	Percentage
Very high	80% and more
High	70–79.9%
Medium	60–69.9%
Low	50–59.9%
Very low	50% and less

## Results

### Research validity and reliability

The developed questionnaire was sent to two experts at Al Quds Open University to check its accuracy and validity. The reliability of the questionnaire was also calculated using the Cronbach alpha coefficient. The results of the Cronbach alpha coefficient are presented in Tables 3–5.

**TABLE 3:** Result of the Cronbach alpha coefficient for the first dimension.

	Participants (*N*)	Questionnaire items (*N*)	Alpha value
Total degree	44	9	0.857

**TABLE 4:** Result of the Cronbach alpha coefficient for the second dimension.

	Participants (*N*)	Questionnaire items (*N*)	Alpha value
Total degree	44	10	0.888

**TABLE 5:** Results of the Cronbach alpha coefficient for the two dimensions.

	Participants (*N*)	Questionnaire items (*N*)	Alpha value
Total degree	44	19	0.935

Tables 3–5 show that the reliability of each domain and the whole questionnaire was 0.857, 0.888, and 0.935, respectively, which is an acceptable reliability index. Obviously, reliability values range between 0.8 and 0.9, indicating that the tools are reliable and that researchers can draw meaningful conclusions from the data and analysis.

To answer the first research question, the researchers measured mean differences and SD differences between repeated measures with the same instrument for each dimension and the total degree to determine the role of the Mentimeter platform in enhancing students’ online engagement in synchronous education (see Tables 6, 7).

**TABLE 6:** Mean and standard deviation of the respondents’ answers (dimension one).

No.	Items	Mean	Std. deviation	Response rate	Impact degree
1	Mentimeter platform is attractive and simple to use.	4.8636	1.40747	81	Very high*
2	Mentimeter’s feature helps me to know the opinion of my students.	4.8864	1.38456	81.3	Very high*
3	Mentimeter presentation software is user-friendly and visually appealing.	5.4091	0.92304	90.2	Very high*
4	On mentimeter platform, various forms of questions foster active discussion.	5.3636	1.01365	89.3	Very high*
5	Word cloud question form helps students express their answers without being afraid of getting embarrassed.	5.1364	1.09100	85.5	Very high*
6	Mentimeter App helps students review content through quizzes, videos, and documents.	5.3864	1.01651	89.6	Very high*
7	Mentimeter enables me to use various slide types to show the presentation’s material.	5.2500	1.16389	87.5	Very high*
8	Mentimeter presentation provides an integrative way of teaching and learning in synchronous environment.	5.2500	1.16389	87.5	Very high*
9	Multiple teaching methodologies that are appropriate for various learning styles are offered by Mentimeter interactive presentation.	5.2955	1.06922	88.2	Very high*
	Total degree	5.2045	0.78382	86.7	Very high*

*Maximum response score is 6.

**TABLE 7:** Mean and standard deviation of the respondents’ answers (dimension two).

No.	Items	Mean	Std. deviation	Response rate	Impact degree
10	Spontaneous participation is facilitated by mentimeter’s quiz feature.	5.0455	1.16048	84	Very high*
11	I can get instant feedback about students’ progress.	5.2045	1.21195	86.7	Very high*
12	Mentimeter platform facilitates students’ engagement on equal terms.	5.1136	1.14559	85.2	Very high*
13	My students easily engage in open-ended discussion.	4.8409	1.25648	80.2	Very high*
14	Mentimeter presentation forms eliminate awkward silences.	4.8864	1.43407	81.3	Very high*
15	Mentimeter enhanced learning by facilitating two-way dialogue.	5.0000	1.21999	83.3	Very high*
16	Mentimeter enables more active participation from students in online lectures.	5.2727	1.10735	87.8	Very high*
17	Student’s achievement of the unit learning outcomes was enhanced by the use of mentimeter presentations.	4.8182	1.26257	80.3	Very high*
18	Employing mentimeter competition enables students to stay longer in the online lecture without getting bored.	5.0000	1.25754	83.3	Very high*
19	Mentimeter increases students’ motivation to learn online more.	5.2500	1.03710	87.5	Very high*
	Total degree	5.0432	0.85682	84	Very high*

*Maximum response score is 6.

Table 6 shows that the average response is very high for all items in the first dimension and the total degree. The average response in the sample is located between 81 and 90.2%. These findings show that teachers have extremely positive attitudes toward using the Mentimeter platform in synchronous online instruction. Teachers, for example, agree on the attractiveness and simplicity of Mentimeter presentation to both teachers and students. Also, they agree on the ability of mentimeter to encourage students’ active learning so they can express their answers, review content through quizzes, videos, and documents, using various forms of questions and slide types. Furthermore, they agree that students with different learning styles can easily engage in a Mentimeter presentation due to the platform’s multiple teaching methodologies.

Table 7 shows that the average response is very high for all items in the second dimension and the total degree. The average response in the sample is located between 80.2–87.8%. This result demonstrates that teachers express very positive perspectives toward the role of the Mentimeter Platform in enhancing students’ online engagement in synchronous online teaching. Teachers, for example, agree on the spontaneous participation that is facilitated by Mentimeter’s quiz feature and the instant feedback they can get about students’ progress. They also agree on students’ active engagement through the use of Mentimeter presentations, open-ended discussion, and competition. Furthermore, they agree that students’ motivation toward learning online courses was enhanced due to using Mentimeter’s tool to present online materials that were appropriate for students’ different learning styles.

To answer the second research question, the researchers used One Way ANOVA to calculate the differences in the total degree of the tool based on the academic degree of participants. The results are shown in the following tables (Tables 8, 9).

**TABLE 8:** Mean and standard deviation (based on academic degree).

Dimensions	Academic degree	*N*	Mean	Std. deviation
Dimension 1	BA	9	5.2469	0.30316
	MA	20	5.1667	1.02503
	Ph.D.	15	5.2963	0.49766
	Total	44	5.2045	0.78382
Dimension 2	BA	9	4.8222	0.56740
	MA	20	5.0700	1.09597
	Ph.D.	15	5.1667	0.50871
	Total	44	5.0432	0.85682
Total	BA	9	5.0346	0.40871
	MA	20	5.1183	1.04480
	Ph.D.	15	5.2315	0.48009
	Total	44	5.1239	0.79942

**TABLE 9:** Results of one-way ANOVA (based on academic degree).

Dimensions		Sum of squares	DF	Mean square	*F*	Sig.
Dimension 1	Between groups	0.321	3	0.107	0.164	0.920*
	Within groups	26.097	40	0.652		
	Total	26.418	43			
Dimension 2	Between groups	0.637	3	0.212	0.275	0.843*
	Within groups	30.931	40	0.773		
	Total	31.568	43			
Total	Between groups	0.259	3	0.086	0.127	0.944*
	Within groups	27.221	40	0.681		
	Total	27.480	43			

*Statistically significant at α ≤ 0.05.

Table 8 shows the results of the means and SD differences according to academic degree for all domains. According to the results, the value of the mean in the first domain that discussed teachers’ attitudes toward using the Mentimeter platform in synchronous online teaching achieved the highest mean value of 5.2045 and a standard deviation value of 0.78382. On the other hand, the lowest mean value was 5.0432 in favor of the second dimension, which presented teachers’ perspectives toward the role of the Mentimeter Platform in enhancing students’ online engagement in synchronous online teaching. According to the results of the same domain levels, the highest mean average for the first domain was 5.2963 in favor of the Ph.D education level, and the lowest mean average value was 4.9630 in favor of the other education level. This indicates that teachers who hold a Ph.D. degree have the highest attitudes toward using the Mentimeter platform in synchronous online teaching in comparison with teachers with other education levels. According to the results of the second domain, the highest mean value was 5.1667 in favor of the Ph.D. education level, and the lowest mean value was 4.8222 in favor of the BA education level. Also, the holders of Ph.D. degrees expressed higher perspectives toward the role of the Mentimeter Platform in enhancing students’ online engagement in synchronous online teaching in comparison with BA degree holders. Whereas, the total degree’s highest mean was 5.2315 in favor of the Ph.D. education level and the lowest value was 4.9981 in favor of other education levels. Overall, most of the Ph.D. degree holders show the highest level of agreement on the role of the Mentimeter platform in enhancing online engagement in synchronous online teaching. While the majority of respondents to the other education level variable expressed the least agreement with the Mentimeter platform’s role in enhancing online engagement in synchronous online teaching.

According to Table 9, there were no statistically significant differences between the participants’ academic degree on all dimensions and the overall degree in the contribution of the Mentimeter platform in enhancing students’ online engagement in synchronous online teaching. To test the role of working environment (educational institution), the researchers used Mean and One Way ANOVA test to measure the differences in the total degree of the tool. The outcomes are shown in Tables 10, 11.

**TABLE 10:** Mean and standard deviation (based on educational institution).

Dimensions	Educational institution	*N*	Mean	Std. deviation
Dimension 1	University	15	5.1926	1.13192
	School	15	5.2815	0.35849
	Educational center	7	5.1270	0.71845
	College	7	5.1429	0.75554
	Total	44	5.2045	0.78382
Dimension 2	University	15	5.1867	1.09340
	School	15	5.0467	0.45335
	Educational center	7	4.9286	0.71581
	College	7	4.8429	1.16884
	Total	44	5.0432	0.85682
Total	University	15	5.1896	1.10443
	School	15	5.1641	0.37199
	Educational center	7	5.0278	0.69750
	College	7	4.9929	0.93746
	Total	44	5.1239	0.79942

**TABLE 11:** Results of one-way ANOVA (based on educational institutions).

Dimensions		Sum of squares	DF	Mean square	*F*	Sig.
Dimension 1	Between groups	0.160	3	0.053	0.081	0.970*
	Within groups	26.259	40	0.656		
	Total	26.418	43			
Dimension 2	Between groups	0.682	3	0.227	0.294	0.829*
	Within groups	30.886	40	0.772		
	Total	31.568	43			
Total	Between groups	0.274	3	0.091	0.134	0.939*
	Within groups	27.206	40	0.680		
	Total	27.480	43			

*Statistically significant at α ≤ 0.05.

Table 10 shows the results of means and SD differences according to the working place variable for all domains. Results show that the value of the mean average of the first domain that discussed teachers’ attitudes toward using the Mentimeter platform in synchronous online teaching has the highest mean average value of 5.2045 and a standard deviation value of 0.78382. On the other hand, the lowest mean value was 5.0432 in favor of the second dimension that presented teachers’ perspectives toward the role of the Mentimeter Platform in enhancing students’ online engagement in synchronous online teaching. According to the results of each domain level, the highest mean average for the first domain was 5.2815 in favor of schools, and the lowest mean average value was 5.1270 in favor of educational centers. This result indicates that teachers who work at schools had the highest attitudes toward using the Mentimeter platform in synchronous online teaching in comparison with other teachers who work at educational centers. According to the results of the second domain, the highest mean value was 5.1867 in favor of universities and the lowest mean value was 4.8429 in favor of colleges. This illustrates that teachers who work at universities expressed higher perspectives toward the role of the Mentimeter Platform in enhancing students’ online engagement in synchronous online teaching in comparison with teachers who work at colleges. Whereas the total degree’s highest mean was 5.1896 in favor of universities and the lowest value was 4.9929 in favor of colleges, overall, most university teachers show the highest degree of agreement on the role of the Mentimeter platform in enhancing online engagement in synchronous online teaching. While the majority of college teachers were divided on the role of the Mentimeter platform in increasing online engagement in synchronous online teaching.

Table 11 shows that there were no statistically significant differences at the significant level of α ≤ 0.05 in the role of the Mentimeter platform in enhancing students’ online engagement in synchronous online teaching as attributed to the working place variable on all dimensions and the total degree. To test the most commonly used Mentimeter’s tool variable, the researchers used Mean and One Way ANOVA, and Scheffe’s *Post Hoc* test to indicate the differences in the total degree of the tool. As shown in Tables 12–14.

**TABLE 12:** Mean and standard deviation (most commonly used Mentimeter’s tool).

Dimensions	The most commonly used Mentimeter’s tool	*N*	Mean	Std. deviation
Dimension 1	Word clouds	8	5.2361	0.57716
	Open- ended questions	4	4.5000	2.04979
	Quizzes	3	4.0370	0.73981
	Presentation	7	5.5714	0.28276
	All the above	22	5.3636	0.33277
	Total	44	5.2045	0.78382
Dimension 2	Word clouds	8	5.0625	0.65670
	Open- ended questions	4	4.4750	1.99228
	Quizzes	3	3.6333	1.10151
	Presentation	7	5.5143	0.37607
	All the above	22	5.1818	0.44362
	Total	44	5.0432	0.85682
Total	Word clouds	8	5.1493	0.59771
	Open- ended questions	4	4.4875	2.01134
	Quizzes	3	3.8352	0.89307
	Presentation	7	5.5429	0.26287
	All the above	22	5.2727	0.34569
	Total	44	5.1239	0.79942

**TABLE 13:** Results of one-way ANOVA (based on most commonly used Mentimeter’s tool).

Dimensions		Sum of squares	DF	Mean square	*F*	Sig.
Dimension 1	Between groups	7.582	4	1.895	3.924	0.009*
	Within groups	18.837	39	0.483		
	Total	26.418	43			
Dimension 2	Between groups	9.234	4	2.308	4.031	0.008*
	Within groups	22.334	39	0.573		
	Total	31.568	43			
Total	Between groups	8.324	4	2.081	4.236	0.006*
	Within groups	19.157	39	0.491		
	Total	27.480	43			

*Statistically significant at α ≤ 0.05.

**TABLE 14:** Results of Scheffe’s *post hoc* test (based on most commonly used Mentimeter’s tool).

Dependent variable	(I) Use Mentimeter	(J) Use Mentimeter	Mean difference (I-J)
Dimension 1	Presentation	Quizzes	1.53439*
Dimension 2	Presentation	Quizzes	1.88095*
Total	Presentation	Quizzes	1.70767*

*Statistically significant at α ≤ 0.05.

Table 12 shows the results of mean and SD differences according to the most commonly used Mentimeter’s tool variable for all domains. Results show that the value of the mean average of the first domain that discussed teachers’ attitudes toward using the Mentimeter platform in synchronous online teaching has the highest mean average value of 5.2045 and a standard deviation value of 0.78382. On the other hand, the lowest mean value was 5.0432 in favor of the second dimension that presented teachers’ perspectives toward the role of the Mentimeter Platform in enhancing students’ online engagement in synchronous online teaching. According to the results of each domain level, the highest mean average for the first domain was 5.5714 in favor of presentations, and the lowest mean average value was 4.0370 in favor of quizzes. This result indicates that teachers who used presentations as an interactive tool in synchronous online teaching have the highest attitudes toward using the Mentimeter platform in synchronous online teaching in comparison with teachers who used quizzes. According to the results of the second domain, the highest mean value was 5.5143 in favor of presentation tools and the lowest mean value was 3.6333 in favor of quizzes. This illustrates that teachers who used presentations in synchronous teaching settings expressed higher perspectives toward the role of the Mentimeter Platform in enhancing students’ online engagement in synchronous online teaching in comparison with teachers who used quizzes. Whereas, the total degree’s highest mean was 5.5429 in favor of presentations, and the lowest value was 3.8352 in favor of quizzes. Overall, most teachers who used presentation tools in synchronous online teaching agreed the most on the role of the Mentimeter platform in increasing online engagement. While the majority of teachers who used quizzes in synchronous teaching had the lowest level of agreement with the Mentimeter platform’s role in increasing students’ online engagement.

Table 13 shows that there were statistically significant differences on the first and second dimensions and the total degree, and so there are statistically significant differences at the level of significance α ≤ 0.05 in the role of the Mentimeter platform in enhancing students’ online engagement in synchronous teaching as attributed to the most commonly used Mentimeter tool variable. To find differences between levels, the researcher used Scheffe’s test for dimensional comparisons between levels to find out between which levels were the differences on the third dimension (Table 10).

Table 14 shows that the differences were on the first and second dimensions, and the total degree between presentations and quizzes was in favor of presentations in the sense that teachers who used presentations have indicated higher attitudes and perspectives toward the role of the Mentimeter platform in enhancing students’ online engagement in synchronous teaching settings than teachers who used quizzes. While the other comparisons are not statistically significant. To answer the third research question, the researchers used the Pearson Correlation Test to find out the correlation between teachers’ attitudes toward using the Mentimeter platform in synchronous online teaching and their perspectives toward the platform’s role in enhancing students’ online engagement in synchronous teaching settings, as shown in Table 15.

**TABLE 15:** Results of Pearson correlation test.

Dimensions	Mean	Std. deviation	Pearson correlation value	Significance value
Teachers’ attitudes	5.2045	0.78382	0.899	0.000*
Teachers’ perspectives	5.0432	0.85682		

*Statistically significant at level α ≤ 0.05.

Table 15 shows that there is a strong positive correlation between teachers’ attitudes toward using the Mentimeter platform in synchronous online teaching and their perspectives toward the platform’s role in enhancing students’ online engagement in synchronous teaching settings. where the value of the coefficient of the Pearson Correlation Test (*r*) was 0.899 since it was greater than 0.5 and the significance value was 0.000, and so there is a positive relationship at the level of significance α ≤ 0.05 between teachers’ attitudes toward using the Mentimeter platform in synchronous online teaching and their perspectives toward the Mentimeter platform’s role in enhancing students’ online engagement in synchronous teaching settings in favor of teachers’ attitudes.

There is a strong positive linear relationship between two continuous dependent variables, as shown in Supplementary Figure 4, and there are differences in their relationship in favor of teachers’ attitudes toward using the Mentimeter platform in synchronous online teaching because its mean average is higher than teachers’ perspectives toward the Mentimeter platform’s role in enhancing students’ online engagement variable. This result also implies that teachers’ positive attitudes toward using such online tools in synchronous online classes assist them in carrying out synchronous course teaching with acceptable quality and facilitate their job in maintaining students’ online engagement.

## Discussion

The present research set out to evaluate the Palestinian teachers’ attitudes toward the Mentimeter Platform and its impact on students’ engagement in online synchronous classes. The results of data analysis demonstrated that the majority of educators perceived that the Mentimeter platform is useful for increasing students, engagement in online synchronous courses. This result seems to be consistent with those of some previous studies (e.g., Vallely and Gibson, 2018; Moorhouse and Kohnke, 2020; Demirci et al., 2021; Sari, 2021; Mohin et al., 2022; Utomo and Utama, 2022). The results of our study also indicated that the attitudes of the participants were favorable toward the usage of the Mentimeter platform in synchronous online instruction. However, we note that these positive perspectives would enable educators to utilize most of the Mentimeter in synchronous learning environments. The findings from this study are consistent with those from Vallely and Gibson (2018), Andriani et al. (2019), Puspa and Imamyartha (2019), Anggriani et al. (2022). In this regard, the study by Quiroz Canlas et al. (2020) focuses on a number of advantages of integrating the Mentimeter into the computer science lecture, including the application’s ease of use, increased participation in class, freedom to express oneself without worrying about coming off as stupid, remembering earlier topics, remembering the key points of the conversation, overcoming boredom, and providing instantaneous feedback on learning. All of these have also been noted by the teachers who participated in our study. The educators have overwhelmingly positive opinions about the Mentimeter Platform’s contribution to increasing students’ online engagement in synchronous online teaching in terms of avoiding boredom. Teachers, for instance, agree that using Mentimeter competitions encourages students to stay in the online lecture for longer periods of time without getting bored and that Mentimeter presentation styles prevent awkward silences.

In addition, we must stress that the average response for all inquiries about teachers’views of the Mentimeter Platform’s contribution to improving students’ online engagement in synchronous education was very high and ranged between 80.2 and 87.8%. Furthermore, when compared to college instructors, the majority of university instructors have the highest level of agreement on the Mentimeter platform’s role in strengthening online engagement in synchronous online teaching, as well as the highest level of agreement among instructors who use presentation tools in such instruction. In this case, to increase students’ involvement in synchronous learning environments, it is necessary to regularly train the teaching staff of schools, learning institutions, and colleges in the use of Mentimeter. As we have previously stated, our goal is to further explore the insights and perspectives surrounding the Mentimeter Platform’s contribution to improving online student engagement in synchronous instruction. The constructive viewpoints of the participants should be highlighted. As a result, it is evident that there is a significant positive association between teachers’ attitudes toward using the Mentimeter platform for synchronous online teaching and their perceptions of the platform’s contribution to increasing students’ online engagement in such circumstances. This indicates that as teachers’ attitudes improved, so did their opinions on how the Mentimeter platform might improve students’ online involvement in real-time learning environments. These outcomes are consistent with earlier research (e.g., Ahmad and Subekti, 2021; Mohin et al., 2022).

On the other hand, students’ point of view about the efficiency of the Mentimeter platform in synchronous online learning was carried out by Puspa and Imamyartha (2019), Demirci et al. (2021), Sari (2021), Mohin et al. (2022), and Samad and Munir (2022), who revealed that students had positive perceptions toward the implementation of Mentimeter in the EFL classroom and there is a significant impact of this online tool in students’ engagement, makes them like the class, motivates them, aids in their ability to concentrate and encourage them to take part in learning. Therefore, synchronous learning integrated with online tools becomes very much needed to be carried out on a sustainable basis.

Finally, it is worth noting that the results of this research are subject to at least three important limitations. First and foremost, this study was completely conducted in Palestine, which is an Asian country. Second, this study was solely focused on the role of the Mentimeter platform in improving students’ engagement. It would be interesting to examine the impact of other online platforms such as Kahoot, Plickers, GoSoapBox, and Poll Everywhere on students’ engagement. Third, in this research, students’ perceptions toward the use of the Mentimeter platform in online synchronous courses were neglected. To attain more comprehensive outcomes, future studies are recommended to simultaneously concentrate on teachers’ and students’ viewpoints.

## Conclusion

The present inquiry sought to delve into Palestinian teachers’ viewpoints regarding the use of the Mentimeter platform in synchronous education. Moreover, it aimed to assess teachers’ perceptions of the role of this platform in enhancing students’ engagement. The results evinced that the majority of participants had positive perceptions toward Mentimeter platform. Moreover, the participants perceived the Mentimeter platform to be highly influential in increasing students’ engagement in online synchronous courses. The results of the current study may have important implications for all teachers who are struggling with student disengagement in online educational environments. Given that the Mentimeter platform serves an important role in improving students’ engagement, teachers are strongly recommended to employ this platform with a view to enhance their students’ engagement. The study’s findings could be useful for teacher educators as well. Considering the positive impact of the Mentimeter platform on students’ engagement, teacher educators need to teach their teacher students how to work with such technologies in educational settings.

## Data availability statement

The original contributions presented in this study are included in the article/Supplementary material, further inquiries can be directed to the corresponding author.

## Ethics statement

Ethical review and approval was not required for the study on human participants in accordance with the local legislation and institutional requirements. Written informed consent for participation was not required for this study in accordance with the national legislation and the institutional requirements.

## Author contributions

AT: conceptualization, methodology, validation, formal analysis, investigation, resources, and writing—original draft preparation. JM: writing—review and editing and supervision. Both authors have read and agreed to the published version of the manuscript, listed have made a substantial, direct, and intellectual contribution to the work, and approved it for publication.

## References

[B1] Abrami P. C. Bernard R. M. Bures E. M. Borokhovski E. Tamim R. M. (2011). Interaction in distance education and online learning: Using evidence and theory to improve practice. *J. Comput. High. Educ.* 23 82–103. 10.1007/s12528-011-9043-x

[B2] Ahmad Z. M. Subekti F. E. (2021). Implementation of problem-based learning in basic statistical concepts using web-based apps. *Int. J. Manag. Hum.* 5 1–4. 10.35940/ijmh.L1360.0851221

[B3] Andriani A. Dewi I. Sagala P. N. (2019). Development of blended learning media using the mentimeter application to improve mathematics creative thinking skills. *J. Phys. Conf. Ser.* 1188:012112. 10.1088/1742-6596/1188/1/012112

[B4] Anggriani M. D. Haryanto H. Atmojo S. E. (2022). The impact of problem-based learning model assisted by Mentimeter Media in science learning on students’ critical thinking and collaboration skills. *Int. J. Elementary Educ.* 6 350–359. 10.23887/ijee.v6i2.46837

[B5] Banna J. Lin M. F. G. Stewart M. Fialkowski M. K. (2015). Interaction matters: Strategies to promote engaged learning in an online introductory nutrition course. *J. Online Learn. Teach.* 11 249–261. 27441032 PMC4948751

[B6] Bower M. Dalgarno B. Kennedy G. E. Lee M. J. Kenney J. (2015). Design and implementation factors in blended synchronous learning environments: Outcomes from a cross-case analysis. *Comput. Educ. J.* 86 1–17. 10.1016/j.compedu.2015.03.006

[B7] Britt M. (2015). How to better engage online students with online strategies. *Coll. Stud. J.* 49 399–404.

[B8] Bundick M. J. Quaglia R. J. Corso M. J. Haywood D. E. (2014). Promoting student engagement in the classroom. *Teach. Coll. Record* 116 1–34.

[B9] Cain W. (2015). “Technology navigators: An innovative role in pedagogy, design and instructional support,” in *Educational innovations and contemporary technologies: Enhancing teaching and learning*, eds Redmond P. Lock J. Danaher P. (London: Palgrave Macmillan), 21–35.

[B10] Carolan F. Kyppö A. (2015). “Teaching process writing in an online environment,” in *Voices of pedagogical development – Expanding, enhancing and exploring higher education language learning*, eds Jalkanen J. Jokinen E. Taalas P. (Dublin: Research-publishing.net), 13–30. 10.14705/rpnet.2015.000285

[B11] Compton M. Allen J. (2018). Student response systems: A rationale for their use and a comparison of some cloud based tools. *Compass J. Learn. Teach.* 11:14. 10.21100/compass.v11i1.696

[B12] Demirci C. Duraklı H. Güdücü A. (2021). The use of audience response system in English teaching: The example of mentimeter/ingilizce öğretiminde dinleyici yanit sistemi kullanimi: Mentimeter örneği. *Eur. J. Educ. Stud.* 8:4083. 10.46827/ejes.v8i12.4083

[B13] Derakhshan A. Fathi J. Pawlak M. Kruk M. (2022). Classroom social climate, growth language mindset, and student engagement: The mediating role of boredom in learning English as a foreign language. *J. Multiling. Multicult. Dev.* 1–19. 10.1080/01434632.2022.2099407

[B14] Derakhshan A. Kruk M. Mehdizadeh M. Pawlak M. (2021). Boredom in online classes in the Iranian EFL context: Sources and solutions. *System* 101:102556. 10.1016/j.system.2021.102556

[B15] Firman F. Rahayu S. (2020). Pembelajaran online di tengah pandemic covid-19. *Indones. J. Educ. Sci.* 2 81–89.

[B16] Grant M. M. Cheon J. (2007). The value of using synchronous conferencing for instruction and students. *J. Interact. Online Learn.* 6 211–226. 10.2196/jmir.9.5.e39 18166527 PMC2270418

[B17] Herawati R. Tjahjono H. K. Qamari I. N. Wahyuningsih S. H. (2022). Teachers’ willingness to change in adapting to Online learning during the covid-19 pandemic. *Cakrawala Pendidikan J. Ilmiah Pendidikan* 41 425–436. 10.21831/cp.v41i2.43233

[B18] Iqbal S. Ahmad S. Akkour K. AlHadab F. AlHuwaiji S. Alghamadi M. (2021). Audience response system (ARS), A way to foster formative assessment and motivation among medical students [version 1]. *MedEdPublish* 10:120. 10.15694/mep.2021.000120.1 38486588 PMC10939530

[B19] John I. (2018). *Mentimeter*. *School librarian*, Vol. 66. 153. Available online at: https://go.galegroup.com/ps/anonymous?id=GALE%7CA555409973&sid=googleScholar&v=2.1&it=r&linkaccess=abs&issn=00366595&p=AONE&sw=w (accessed February 4, 2023).

[B20] Lapshova A. V. Vaganova O. Ivanov V. Bulaeva M. Kutepov M. (2021). Ways of conducting surveys in the student environment in remote format. *Eduweb Mag.* 15 225–234. 10.46502/issn.1856-7576/2021.15.03.19

[B21] Little C. (2019). Mentimeter smartphone student response system: A class above clickers. Compass. *J. Learn. Teach.* 9 1–3. 10.21100/compass.v9i13.328

[B22] Madiseh F. R. Al Abri A. Sobhanifar H. (2022). Integration of mentimeter into the classroom: A scoping review. *Res. Square* [Preprint]. 10.21203/rs.3.rs-1339347/v1

[B23] Martin F. Bolliger D. U. (2018). Engagement matters: Student perceptions on the importance of engagement strategies in the online learning environment. *Online Learn. J.* 22 205–222. 10.1186/s12913-016-1423-5 27409075 PMC4943498

[B24] Mohin M. Kunzwa L. Patel S. (2022). Using mentimeter to enhance learning and teaching in a large class. Preprint. *Int. J. Educ. Policy Res. Rev.* 9 48–57. 10.15739/IJEPRR.22.005

[B25] Moorhouse B. L. Kohnke L. (2020). Using mentimeter to elicit student responses in the EAP/ESP classroom. *RELC J.* 51 198–204. 10.1177/0033688219890350

[B26] Morrison J. D. (2015). *The effects of electronic response systems on student learning. Ph.D. thesis.* Kalamazoo, MI: Western Michigan University.

[B27] Pawlak M. Derakhshan A. Mehdizadeh M. Kruk M. (2021). Boredom in online English language classes: Mediating variables and coping strategies. *Lang. Teach. Res.* 13621688211064944. 10.1177/13621688211064944

[B28] Pichardo J. I. López-Medina E. F. Mancha-Cáceres O. González-Enríquez I. Hernández-Melián A. Blázquez-Rodríguez M. (2021). Students and teachers using mentimeter: Technological innovation to face the challenges of the COVID-19 pandemic and post-pandemic in higher education. *Educ. Sci.* 11:667.

[B29] Pikhart M. Al-Obaydi L. H. Rehman M. A. (2022a). A quantitative analysis of the students’ experience with digital media in L2 acquisition. *Psycholinguistics* 31 118–140. 10.31470/2309-1797-2022-31-1-118-140

[B30] Pikhart M. Klimova B. Al-Obaydi L. H. Dziuba S. Emerych A. C. (2022b). The quantitative evaluation of subjective satisfaction with digital media in L2 acquisition in younger adults: A study from Europe, Asia, and Latin America. *Front. Psychol.* 13:946187. 10.3389/fpsyg.2022.946187 36467196 PMC9714452

[B31] Puspa A. Imamyartha D. (2019). Experiences of social science students through online application of mentimeter in English milieu. *IOP Conf. Ser. Earth Environ. Sci.* 243:012063.

[B32] Quang P. (2018). “Utilizing electronic devices in English-speaking Vietnamese university classrooms,” in *Proceedings of the 5th international foreign language learning and teaching conference*, Zurich, 121–129.

[B33] Quiroz Canlas F. Nair S. Nirmal Doss A. (2020). “Mentimeter app in computer science courses: Integration model and students’ reception,” in *Proceedings of the 2020 12th international conference on education technology and computers*, London, 1–5. 10.1145/3436756.3436757

[B34] Ramsey D. Evans J. Levy M. (2016). Preserving the seminar experience. *J. Polit. Sci. Educ.* 12 256–267.

[B35] Revere L. Kovach J. V. (2011). Online technologies for engaged learning: A meaningful synthesis for educators. *Q. Rev. Distance Educ. J.* 12 113–124. 27606391

[B36] Rusakova O. Tamozhska I. Tsoi T. Vyshotravka L. Shvay R. (2022). Scientific and communicative interaction in HEIs during pandemic. *Int. J. Health Sci.* 6 115–124.

[B37] Samad P. Munir F. S. (2022). The utilizing of mentimeter platform in enhancing the EFL students’ English skills in digital era. *Edumaspul J. Pendidikan* 6 1645–1650. 10.33487/edumaspul.v6i2.4380

[B38] Sari A. (2021). The impacts of mentimer-based activities on EFL students’ enagement in Indonesia. *J. Lang. Lang. Teach.* 24 249–260. 10.24071/llt.v24i1.3025

[B39] Utomo P. Utama M. (2022). Enhancing students’ engagement using interactive applications in online lectures. *Indones. J. Med. Educ.* 11 326–331.

[B40] Vallely K. Gibson P. (2018). Engaging students on their devices with mentimeter. *Compass J. Learn. Teach.* 11 1–6.

[B41] Weitze C. L. (2015). “Pedagogical innovation in teacher teams: An organizational learning design model for continuous competence development,” in *Proceedings of the 14th European conference on e-Learning ECEL-2015*, eds Jeferies I. A. Cubric M. (Hatfield), 629–638.

[B42] Weitze C. L. Orngreen R. Levinsen K. (2013). “The global classroom video conferencing model and first evaluations,” in *Proceedings of the on E-Learning: SKEMA Business School*, eds Ciussi I. M. Augier M. (Sophia Antipolis), 30–31.

[B43] Wong P. M. Yunus M. M. (2020). Enhancing writing vocabulary using Mentimeter. *Int. J. Learn. Teach. Educ. Res.* 19 106–122. 10.26803/ijlter.19.3.7

