# Supplementary material for: Enhancing EFL students’ engagement in online synchronous classes: The role of the Mentimeter platform
Source: Front Psychol. 2023 Feb 23;14:1127520. doi: 10.3389/fpsyg.2023.1127520 (PMC9996027; doi:10.3389/fpsyg.2023.1127520)
Supplement: Supplementary file 1 [file Data_Sheet_1.docx]

**Appendix**


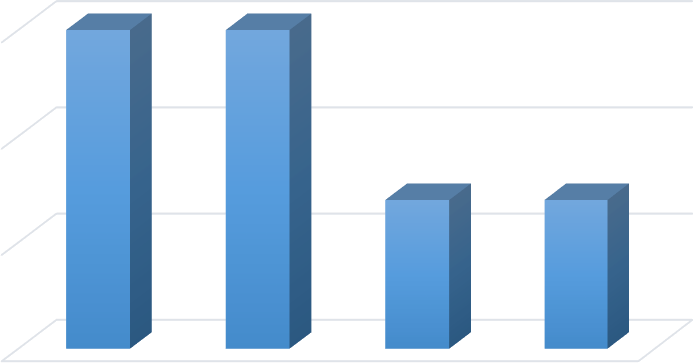


15

15

15

10

7

7

5

0

University

School

Educational Center

College

**Figure 1.** Educational Institution


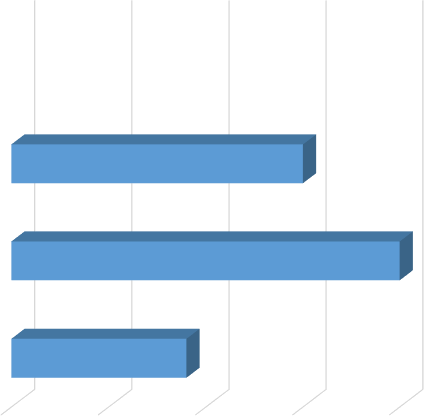


Ph.D.

15

MA

20

BA

9

0

5

10

15

20

**Figure 2.** Academic Degree


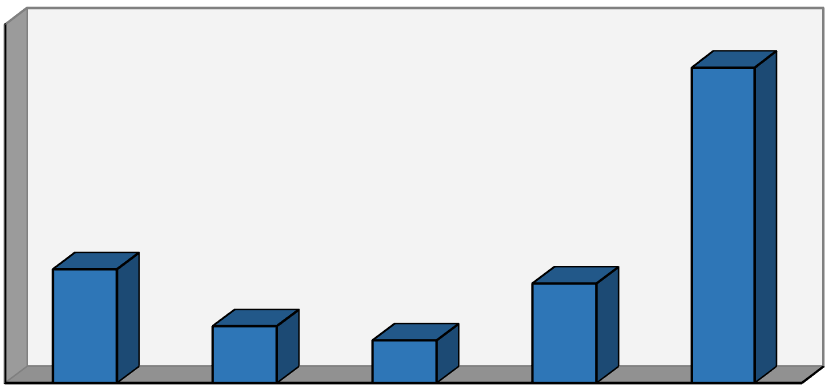


**25**

**22**

**20**

**15**

**Frequency**

**10**

**5**

**0**

**Word clouds Open- ended**

**questions**

**Quizzes**

**Presentation All the above**

**3**

**4**

**7**

**8**

**Figure 3**. The most commonly used Mentimeter’s tool

**Figure 4**. Results of Pearson Correlation Test
